# Development of Gluten-Free Bread Using Teosinte (*Dioon mejiae*) Flour in Combination with High-Protein Brown Rice Flour and High-Protein White Rice Flour

**DOI:** 10.3390/foods12112132

**Published:** 2023-05-25

**Authors:** Franklin Delarca Ruiz, Ricardo S. Aleman, Shirin Kazemzadeh Pournaki, Mallerly Sarmiento Madrid, Andrea Muela, Yeimi Mendoza, Jhunior Marcia Fuentes, Witoon Prinyawiwatkul, Joan M. King

**Affiliations:** 1 Faculty of Technological Sciences, Universidad Nacional de Agricultura, Catacamas 16201, Honduras; fdelarca19a0218@unag.edu.hn (F.D.R.); msarmiento19a0164@unag.edu.hn (M.S.M.); jmarcia@unag.edu.hn (J.M.F.); 2 School of Nutrition and Food Sciences, Louisiana State University Agricultural Center, Baton Rouge, LA 70803, USA; rsantosaleman@lsu.edu (R.S.A.); amuela1@lsu.edu (A.M.); ymendoza@agcenter.lsu.edu (Y.M.); wprinyawiwatkul@agcenter.lsu.edu (W.P.); 3 Department of Dairy & Food Science, South Dakota State University, Brookings, SD 57007, USA; shirin.kazemzadehpournaki@sdstate.edu

**Keywords:** teosinte, rice, gluten free, bread, mixture design

## Abstract

Gluten-free bread is an important product that is under development using different sources, such as rice and starchy plants. Teosinte seeds are utilized by ethnic groups in Honduras to produce gluten-free flour to prepare traditional baked goods and beverages. The quality of gluten-free products could vary depending on flour properties, such as amylose content, particle size, and water absorption capacity. A good strategy for developing baked goods is to mix different cereal grain sources to optimize their physicochemical properties. As a result, the current study aimed to develop bread from novel flours including teosinte (TF), high-protein brown rice (BRF), and high-protein white rice (WRF). Breads were analyzed for hardness, specific volume, and color utilizing a Simplex-Centroid mixture design coupled with the desirability function. Pasting, and rheological characteristics of the flours, were also analyzed. For flour characteristics, TF addition to BRF or WRF decreased the peak, trough, breakdown, setback, and final viscosities, which would result in a more stable bread and decrease the flow index of rice flour dispersions. BRF and WRF had similar pasting properties, except that BRF had a lower breakdown viscosity. For bread characteristics, TF addition to BRF or WRF increased the specific volume and hardness of the bread compared to rice flour alone. L* of the crust and crumb a* values were increased with greater TF in the mixture, whereas TF decreased the crust a*and b* values and crumb L* values when mixed with BRF or WRF compared to rice flours alone. WRF and BRF were similar in crumb color (L* and a*), except that BRF had greater crumb yellowness (b*). Teosinte flour can be used in combination with rice flour to produce bread with good quality.

## 1. Introduction

Bread uses the ability of hydrated gluten to build a viscoelastic network [1], which causes gas to be trapped, and thus increases its volume. Additionally, gluten in bread plays a key role in moisture control [2]. It is a high-molecular weight protein found in the endosperm of cereals including wheat, barley, and rye. Additionally, it is a storage protein in a group of flowering plants utilized during the growth and germination process that consists of two types of proteins, i.e., a glutenin and a prolamin (gliadin found in wheat), which can be broken down to produce α, β, and γ peptides [3].

However, gluten in bread can cause problems in a group of consumers. Celiac disease is an autoimmune disorder seen in people who genetically have the potential to develop an immune reaction to gluten. The first place affected by this disease is the small intestine; nevertheless, it has a wide distribution that includes both intestinal and extra-intestinal symptoms [4]. Currently, the most effective and safest treatment for people affected by Celiac disease is to use a gluten-free (GF) diet, which causes an improvement of the small intestinal mucosa [5,6].

Gluten helps by forming a sticky, elastic dough with gas retention, as well as shaping the structure [7]. In the absence of gluten in bread, the ability of the bread to hold carbon dioxide produced by yeast is significantly reduced, which results in bread with a firm texture, as well as low specific volume [8].

Gluten-free bread available in the market is usually obtained by replacing wheat flour with rice flour with or without corn starch. Rice flour is known for its low price and mild taste, as well as antiallergenic properties, which is why it is utilized in these products. The rice flour used to prepare gluten-free bread is mostly from white rice [9]. However, gluten-free bread, which is obtained by using white rice flour, is nutritionally imbalanced due to the removal of the entire bran layer, leaving mainly starch and protein. It is expected that the use of brown rice flour in gluten-free bread will compensate for this deficiency because brown rice has non-starch nutrients including dietary fiber, minerals, and bioactive compounds in its bran layer [10].

Teosinte (*Dioon mejiae*) as an endemic tree in Honduras is one of the dioecious trees and belongs to the category of minor cereals. Minor cereals occur only in a few parts of the world, that is why their use on a large scale is not common. In addition to Teosinte, other plants including teff, millet (pearl, proso, finger, foxtail, and Kodo), fonio (black and white), jungle rice, and Job’s tears are in this category [11,12,13,14]. Teosinte seeds are locally used in the preparation of flour as well as other traditional foods and drinks. The nutritional value of teosinte seed is high with protein and methionine levels higher than maize. However, no difference was reported in the amino acids, such as lysine, tryptophan, or niacin [15]. In the distant past, regional people used the seeds of this plant after washing and drying to prepare foods such as bread, donuts, tamales, and tortillas. Additionally, a type of starch called sago is obtained from this plant, which is used as a food supplement by the natives of that area [16].

Some studies have been conducted in the field of gluten-free bread production and improvement. Some innovative technologies were proposed to improve quality, replace or imitate the gluten network by using exogenous substances including hydrocolloids, emulsifiers, proteins, and cross-linking enzymes [6,17]. As mentioned previously, among the available approaches for gluten-free bread production is the use of alternative plant sources for wheat. Different types of flour and starch (rice, corn, cassava, soybean, and peanut) have been used to produce gluten-free bread [18,19]. For example, active soybean flour improved the volume and structure of gluten-free bread [19]. Bread made with corn flour and chickpea flour became softer with greater levels of chickpea flour, which was thought to be due to greater protein levels [20]. Other flours are used to increase the nutritional quality of gluten free breads [21].

In this regard, due to the essential need to diversify the diet and for those who have special nutritional requirements, such as celiac patients, it is mandatory to provide an innovative diet. For this purpose, examining the potential of traditional and lesser-known food sources as alternatives and the expansion of their use to produce innovative gluten-free foods that are acceptable to consumers seems promising. Due to the potential for using brown rice flour and teosinte flour in gluten-free foods, and the few studies in this field, this study aims to use these plant resources to develop gluten-free bread.

## 2. Material and Method

### 2.1. Experimental Design

Table 1 shows the treatment design for bread production using a Simplex-Centroid Mixture Design (CSMD). The independent variables were the proportions of high-protein brown rice flour (Cahokia), high-protein white rice flour (Cahokia), and teosinte flour, while the dependent variables included specific volume (g/mL), color (L*, b* and a*) and texture (resilience, cohesiveness, hardness, and springiness). The obtained response from each investigated parameter was analyzed using adjusting the cubic model (Y = β1 × 1 + β2 × 2 + β3 × 3 + β12 × 1 × 2 + β13 × 1 × 3 + β23 × 2 × 3 + β123 × 1 × 2 × 3) at *p* < 0.05 and using regression to determine significant differences in parameters for the level of flour/starch used (dependent variables). The bread formula was optimized using the desirability function methodology (DOM) [22]. The objective of the mixture design is to optimize flour concentrations of high-protein brown rice flour (Cahokia), high-protein white rice flour (Cahokia), and teosinte flour regarding physicochemical characteristics.

### 2.2. Preparing the Bread

The amounts of each ingredient used are shown in Table 2 for gluten-free breads and control breads (the same procedure was used for both type of breads and only the formulation varied). To make the bread, Fleischmann’s activated dry yeast (ACH Food Companies, Inc., Memphis, TN, USA) was mixed with sugar (Great Value, Leander, TX, USA) and distilled water, then rested for 42 h. Subsequently, by using a Globe stand mixer (model SP5 Global Food Equipment, Dayton, OH, USA), the other dry ingredients (Great Value, Leander, TX, USA) were mixed. The mixture was gently stirred for 30 s. Next, in a separate container, room temperature eggs (Great Value, Leander, TX, USA), vegetable oil (Great Value, Leander, TX, USA), and apple cider vinegar (Great Value, Leander, TX, USA) were weighed and added to the dry ingredients and gently mixed for 1 min. Then, the yeast mixture was gradually added and mixed for 7 min. In the production process of gluten-free bread, the consistency is usually similar to batter (instead of dough, which can be kneaded). Vegetable oil was sprayed in a mini loaf pan (15.4 × 8.6 × 4.7 cm) to grease. Next 150 g of standard-loaf batter was weighed in the pan and the surface was smoothed with a spatula. The relative humidity and temperature of a full-size Metro proofing cabinet (C599-SDS-U Intermetro Industries Corporation, Wilkes-Barre, PA, USA) were set to 90% and 100 °F, respectively, then the pan was placed in the cabinet for 30 min. Afterwards the pan was placed in the center of a Baxter mini-rotating rack gas oven (model OV310G) at a temperature of 345 °F and baked for 20 min. At the end of the baking process, the bread was left in the pan for 5 min to cool and then removed from the pan. After an hour of cooling, a sanitized, electric, meat-slicing machine (model S-4 Sanitary Scale Company, Belvidere, IL, USA) was used to prepare slices of 2.5 cm for color and texture analysis.

### 2.3. Flour Rheological and Pasting Properties

The pasting properties of the flours were evaluated based on the AACC method 61.02.01 [23] by using a Rapid Visco Analyzer (RVA) (RVA-4, Newport Scientific Pty. Ltd., Warriewood, Australia). Rheological properties were evaluated with a rheometer (AR 2000ex, TA Instruments, New Castle, DE, USA) by parallel disc geometry and 40 mm dimensions with a gap of 3 mm. Dispersions of 5% *w*/*w* were stirred at medium speed for 15 min and heated for 30 min. Instantly, hot paste (1 mL) was placed in the rheometer. When the sample temperature reached 25 °C, rheological analysis was performed with two types of evaluation (steady shear flow as well frequency sweep) using the method from Ye et al. [24] with slight modifications.

### 2.4. Bread Physical Features

The specific volume of bread (mL/g) was measured according to the AACC method 10-05 [25] with rapeseed. The texture of the sample was analyzed with a texture analyzer (Texture Technologies Corporation, T.A. XT plus, Scarsdale, NY, USA) based on the AACC method 74-09 (2000) using a two-inch cylinder probe. The bread was cut into to 2.5 cm width slices to examine the bread’s firmness. The parameters were set to a 40% compression at a rate of 1.7 mm/s. With colorimeter equipment (Konica Minolta BC-10 Baking Contrast Meter, Wayne, NJ, USA), L* (brightness/darkness), a* (redness/greenness), and b* (yellow/bluish) samples were analyzed in triplicate.

### 2.5. Statistical Analysis

For the simplex-centroid design, generation of the corresponding response surfaces and coefficients of the special cubic model was performed in the Minitab 17 program (2014, Minitab LLC, State College, PA, USA) to check the characteristics of bread. One-way analysis of variance (ANOVA) and Tukey’s post-hoc test were conducted.

## 3. Results and Discussion

### 3.1. Bread Characteristics

**Figure 1:**
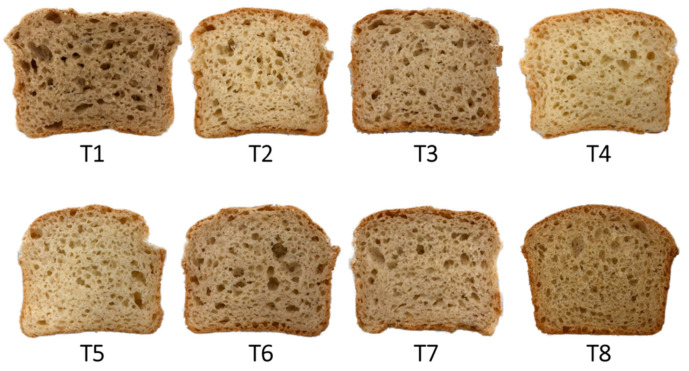
Appearance of central slices of breads crumbs. (T1 = TF = Teosinte Flour, T2 = BRF = High-Protein Brown Rice, T3 = WRF = High-Protein White Rice Flour, T4 = TRF-BRF = Teosinte Flour with High-Protein Brown Rice, T5 = TF-WRF = Teosinte Flour with High-Protein White Rice Flour, T6 = BRF-WRF = High-Protein White Rice Flour with High-Protein Brown Rice Flour, T7 = TF-BRF-WRF = Teosinte Flour with High-Protein White Rice Flour and with High-Protein Brown Rice Flour) Control (T8) = bread made with wheat flour.

Prepared bread samples are shown in Figure 1 compared to control wheat flour bread.

**Figure 2:**
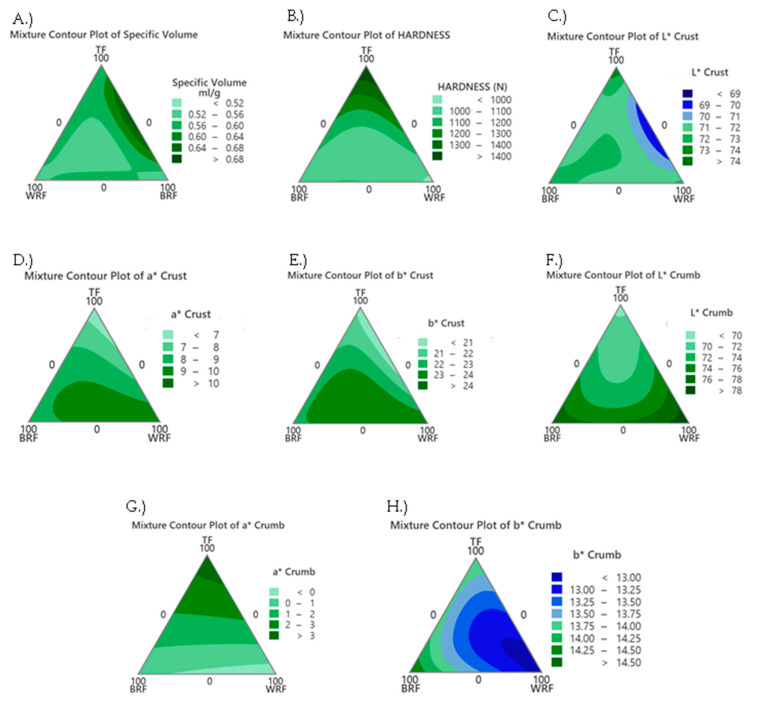
Contour plot of the breads (**A**) Specific Volume, (**B**) Hardness, (**C**) L* Crust, (**D**) a* Crust, (**E**) b* Crust, (**F**) L* Crumb, (**G**) a* Crumb, and (**H**) b* Crumb with *BRF—High-Protein Brown Rice Flour, *TF—Teosinte Flour, and *WRF—High-Protein White Rice.

Figure 2A shows that the bread sample prepared with a combination of TF*BRF presented a greater specific volume compared to BRF and TF individually. Kadan et al. [26] found that rice bread had a lower specific volume than wheat bread; in our study the addition of TF to BRF resulted in a greater specific volume. According to Bastias-Montes et al. [11], protein and total starch contents were 9.67 ± 0.08% and 67.90 ± 0.68% in teosinte flour, and Aleman et al. [27] found that high-protein brown rice flour had 12.2 ± 0.14% protein and 65.4 ± 0.5% starch, while high-protein white rice flour had 10.23 ± 0.26% protein and 75.15 ± 0.20% starch [28]. The specific volume is directly related to water absorption of the network, which affects bread quality, and bread with greater starch content tended to have a greater specific volume [29]. Our study does not show this, as bread with a greater protein, TF*BRF, had a greater specific volume, which could be due to the protein also binding to the water and stabilizing the starch gel [30]. Additionally, the mixture which contained equal percentages of TF, BRF, and WRF showed a lower specific volume similar to bread with WRF or BRF alone. Furthermore, TF alone had the greatest hardness (Figure 2B) compared to other treatments, and Table 3 coefficients indicated that TF had a greater positive effect on the hardness than other flours, which might be due its lower protein content which leads to less water binding and decreased the loaf specific volume [30]. Greater hardness is associated with a lower specific volume, as less water binding could lead to a dryer firmer product with less stable air pockets. WRF alone and BRF alone had the lowest hardness with WRF being lower than BRF. Paz et al. [31] found a similar result for hardness between brown and white rice flour breads.

Crust lightness (L*) was lowest for TF*WRF bread, while TF*WRF*BRF bread had a greater lightness compared to bread made from BRF*TF and BRF*WRF (Figure 2C). Crust redness was the lowest in 100% TF bread (Figure 2D). Bread with TF*WRF and TF*BRF had lower a* compared to BRF*WRF. In another study, the addition of kale to bread decreased a* value due to the addition of the green color [32]. WRF increased the redness in bread which was made from a blend of WRF and BRF. The crust yellowness pattern (Figure 2E) was the same as the redness pattern which means WRF resulted in a greater yellowness in WRF alone, BRF-WRF, and TF-BRF-WRF. TF resulted in a lower yellowness in bread crust for TF alone and TF-WRF.

The crumb color pattern was different from the crust pattern. The lightness of the bread crumb (Figure 2F) was lowest with the use of TF alone, which might be due to the natural, brownish pigments in the seeds [33]. WRF was responsible for lightness in WRF alone, TF-WRF, and BRF-WRF. TF alone had the highest a*, and T_3_ had the lowest redness (Figure 2G) among breadcrumbs. According to Figure 2H, WRF tended to decrease yellowness (b*) in TF-WRF and BRF-WRF. BRF breadcrumb had the greatest yellowness compared to other treatments, which may be due to the presence of yellow pigments [34].

### 3.2. Flour Pasting and Rheological Properties

**Figure 3:**
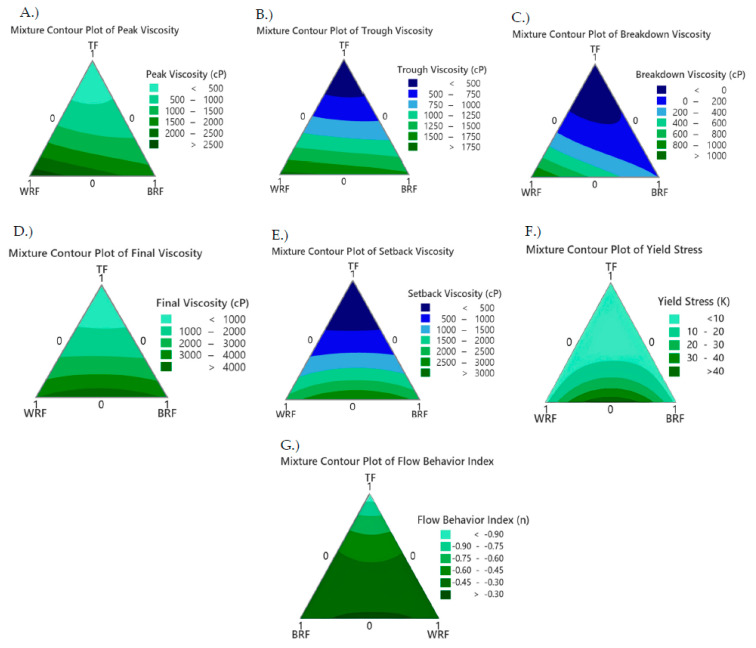
Contour plot of the flour dispersions (**A**) Peak Viscosity, (**B**) Trough Viscosity, (**C**) Breakdown Viscosity, (**D**) Final Viscosity, (**E**) Setback Viscosity, (**F**) Yield Stress, and (**G**) Flow Behavior Index with *BRF—High-Protein Brown Rice Flour, *TF—Teosinte Flour, and *WRF—High-Protein White Rice.

Figure 3 shows peak, trough, breakdown, final, and setback viscosities, as well as yield stress, and flow behavior index with coefficients shown in Table 3. Figure 3A shows that TF alone had the lowest peak viscosity with the lowest coefficient (275) (Table 3) among the flours, while the addition of WRF to the bread blend increased the peak viscosity with a greater coefficient (1739.7), meaning a greater positive effect on peak viscosity (Table 3) than TF. The lower peak viscosity (Figure 3A) of TF resulted in the lowest breakdown viscosity (Figure 3C) coefficient (6.7) (Table 3), and greater past stability compared to BRF and WRF. BRF and WRF showed a greater positive influence on peak, trough final and setback viscosities with greater coefficients than TF (Table 3). Combinations of TF combined with BRF or WRF caused a negative influence resulting in lower pasting viscosities. Other model coefficients did not differ greatly among the individual flours (Table 3). Greater setback viscosity (Figure 2E) in BRF-WRF is associated with a greater retrogradation potential of the starch granules after cooking, which means it might cause a firmer product over time. However, there is no significant correlation between viscosity and starch concentration or distribution of the granules [35].

The combination of the WRF and BRF increased the yield stress and flow behavior index and TF had the lowest yield stress and flow behavior index (Figure 3F,G). According to the current study, all flow behavior indices were less than 1.0, indicating that all pastes exhibited pseudoplastic and shear-thinning behavior. WRF and BRF showed results closest to *n* = 1, which corresponds to a Newtonian fluid [36].

**Figure 4:**
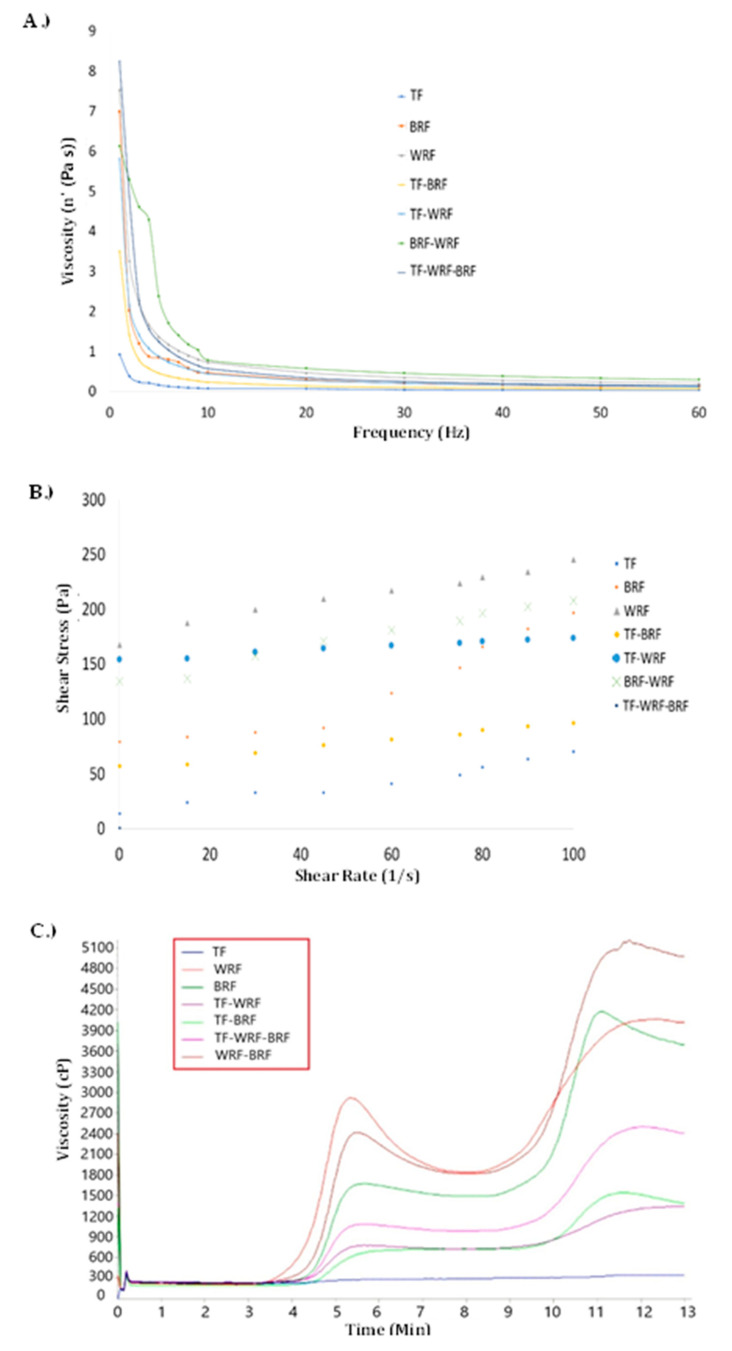
(**A**) steady shear flow measurements, (**B**) frequency sweep analysis, and (**C**) Viscosity profiles of flour dispersions (TF = Teosinte Flour, BR = High-Protein Brown Rice, WF = High-Protein White Rice Flour, TF-BF = Teosinte Flour with High-Protein Brown Rice, TF-WF = Teosinte Flour with High-Protein White Rice Flour, BF-WF = High-Protein White Rice Flour with High-Protein Brown Rice Flour, TF-BF-WR = Teosinte Flour with High-Protein White Rice Flour and with High-Protein Brown Rice Flour).

The complex viscosity (Figure 4A) decreases with increasing frequencies representing a shear-thinning flow behavior. The shear stress as a function of the shear rate is indicated in Figure 4B. which shows all treatments had non-Newtonian behavior due to the increase of the shear stress with the shear rate-like [37] pseudoplastic behavior. BRF-WRF had high-shear stress compared to other treatments. The pasting behavior of treatments is exhibited in Figure 4C, and various parameters were measured like peak, trough, breakdown, final, and setback viscosity. The shape of the pasting curve is different for different flour treatments and significant differences can be observed among GF flours, confirming the contour plot illustrations (Figure 3). The pasting curves of WRF alone had the highest value at peak viscosity and WRF-BRF had the highest final viscosity. The pasting process is the absorption of water by starch granules and granules lose their crystalline structure after swelling properly. According to the steady peak of TF for pasting over time and the heating process, amylose double helices were not melted in the cooking process, granules resisted swelling [38], and granules had a lower rate of water absorption and swelling compared to other samples [39]. According to Table 3, a greater influence was observed for BRF on peak viscosity, as well as a trough, breakdown, and final viscosity, than other single flour samples, while TF had a negative effect on these parameters when mixed with BRF. A higher protein could lead to lower peak viscosity, affecting peak time, trough, and breakdown viscosities by lowering the water-holding capacity of the starch during gelatinization [40]. Peak viscosity, trough viscosity, and breakdown viscosity for WRF were greater than BRF, which may be due to the greater starch content in WRF, 75.15 ± 0.20% vs. 65.4 ± 0.5% for BRF [37].

**Table 3:** Coefficients for the cubic model for bread and flours characteristics.

Response	TF	BRF	WRF	TF-BRF	TF-WRF	BRF-WRF	TF-BRF-WRF	*R* ^2^
Specific Volume (mL/g)	0.01	0.01	0.01	>0.01	>0.01	>0.01	>0.01	87.0%
Hardness (N)	14.9	10.5	9.68	−0.03	0.01	0.03	−0.03	92.8%
L* (Crust)	0.74	0.73	0.72	>−0.01	>−0.01	>−0.01	>0.01	80.9%
a∗ (Crust)	0.06	0.08	0.10	0.01	>−0.01	>0.01	>0.01	95.6%
b∗ (Crust)	0.20	0.22	0.24	>0.01	>−0.01	>0.01	>0.01	87.7%
L* (Crumb)	0.69	0.77	0.79	>−0.01	>−0.01	>−0.01	>−0.01	92.7%
a* (Crumb)	0.03	>0.01	>−0.01	>0.01	>0.01	>−0.01	>−0.01	99.4%
b* (Crumb)	0.14	0.15	0.12	>−0.01	>−0.01	>−0.01	>−0.01	75.5%
Peak Viscosity (cP)	275	2848.3	1739.7	−2909	−1164	−29	−2533	99.2%
Trough Viscosity (cP)	268.3	1811.7	1541.3	−1128	−814	181	−1253	98.6%
Breakdown Viscosity (cP)	6.7	1036.7	198.3	−1781.3	−350	−210	−1280	99.6%
Final Viscosity (cP)	324	3969	3854	−2883	−2805	3370	−7483	98.1%
Setback Viscosity (cP)	56	2158	2312	−1755	−1991	3189	−6230	97.7%
Flow behavior index (n)	0.85	0.31	0.44	1.31	1.27	0.41	1.12	95.8%
Yield Stress (K)	5.72	7.19	6.83	−2.92	3.65	166.90	−367	97.4%

(*p* > 0.05) of the independent variables of the cubic model adjusted for cupcake characteristics (TF = Teosinte Flour, BRF = High-Protein Brown Rice, WRF = High-Protein White Rice Flour, TF-BRF = Teosinte Flour with High-Protein Brown Rice, TF-WRF = Teosinte Flour with High-Protein White Rice Flour, BRF-WRF = High-Protein White Rice Flour with High-Protein Brown Rice Flour, TF-BRF-WRF = Teosinte Flour with High-Protein White Rice Flour and with High-Protein Brown Rice Flour).

## 4. Conclusions

This research examined the development of gluten-free bread by using TF, BRF, and WRF to observe the effects of the different sources on bread properties. Lower pasting properties, such as breakdown and setback viscosity, indicated that TF samples would be more stable. In bread, TF had negative effects on specific volume, texture, and color of breads, resulting in a greater hardness and greater crumb darkness, but these issues can be mitigated by using a combination of brown rice flour or white rice flour with teosinte flour to make the breads.

## Figures and Tables

**Table 1:** Experimental design for bread making with simplex centroid design.

Treatments	*TF	*BRF	*WRF
▪ TF alone	100%	0%	0%
▪ BRF alone	0%	100%	0%
▪ WRF alone	0%	0%	100%
▪ TF-BRF	50%	50%	0%
▪ TF-WRF	50%	0%	50%
▪ BRF-WRF	0%	50%	50%
▪ TF-BRF-WRF	33.337%	33.337%	33.337%

*BRF—High-Protein Brown Rice Flour, *TF—Teosinte Flour, *WRF—High-Protein White Rice.

**Table 2:** Percentages of gluten-free bread ingredients.

Gluten Free Breads		Control Bread	
Ingredients	Percentage	Ingredient	Percentage
Flour *	17.09%	All purpose flour	42.32%
Tapioca flour	14.64%	Whole wheat flour	10.83%
Sugar	3.33%	Sugar	6.66%
Salt	0.98%	Salt	1.50%
Active dry yeast	0.88%	Active dry yeast	1.17%
Water	32.2%	Water	30.67%
Vegetable oil	1.47%	Vegetable oil	4.89%
Vanilla	1.96%	Vanilla	1.96%
Cornstarch	16.11%		
Egg	9.76%		
Xanthan gum	0.78%		
Baking powder	0.49%		
Apple cider vinegar	0.29%		

* Treatments varied only by mixture design treatments illustrated in Table 1. Control bread = wheat bread.

## Data Availability

Data is contained within the article.
